# Individual Shrink Wrapping of Zucchini Fruit Improves Postharvest Chilling Tolerance Associated with a Reduction in Ethylene Production and Oxidative Stress Metabolites

**DOI:** 10.1371/journal.pone.0133058

**Published:** 2015-07-15

**Authors:** Zoraida Megías, Cecilia Martínez, Susana Manzano, Alicia García, María del Mar Rebolloso-Fuentes, Dolores Garrido, Juan Luis Valenzuela, Manuel Jamilena

**Affiliations:** 1 Departamento de Biología y Geología, Agrifood, Campus of International Excellence (ceiA3), Universidad de Almería, La Cañada de San Urbano s/n, 04120 Almería, Spain; 2 Departamento de Agronomía, Agrifood, Campus of International Excellence (ceiA3), Universidad de Almería, La Cañada de San Urbano s/n, 04120 Almería, Spain; 3 Departamento de Fisiología Vegetal, Facultad de Ciencias, Universidad de Granada, Fuentenueva s/n, 18071 Granada, Spain; Zhejiang University, CHINA

## Abstract

We have studied the effect of individual shrink wrapping (ISW) on the postharvest performance of refrigerated fruit from two zucchini cultivars that differ in their sensitivity to cold storage: Sinatra (more sensitive) and Natura (more tolerant). The fruit was individually shrink wrapped before storing at 4°C for 0, 7 and 14 days. Quality parameters, ethylene and CO_2_ productions, ethylene gene expression, and oxidative stress metabolites were assessed in shrink wrapped and non-wrapped fruit after conditioning the fruit for 6 hours at 20°C. ISW decreased significantly the postharvest deterioration of chilled zucchini in both cultivars. Weight loss was reduced to less than 1%, pitting symptoms were completely absent in ISW fruit at 7 days, and were less than 25% those of control fruits at 14 days of cold storage, and firmness loss was significantly reduced in the cultivar Sinatra. These enhancements in quality of ISW fruit were associated with a significant reduction in cold-induced ethylene production, in the respiration rate, and in the level of oxidative stress metabolites such as hydrogen peroxide and malonyldialdehyde (MDA). A detailed expression analysis of ethylene biosynthesis, perception and signaling genes demonstrated a downregulation of *CpACS1* and *CpACO1* genes in response to ISW, two genes that are upregulated by cold storage. However, the expression patterns of six other ethylene biosynthesis genes (*CpACS2* to *CpACS7*) and five ethylene signal transduction pathway genes (*CpCTR1*, *CpETR1*, *CpERS1*, *CpEIN3*.*1* and *CpEN3*.*2*), suggest that they do not play a major role in response to cold storage and ISW packaging. In conclusion, ISW zucchini packaging resulted in improved tolerance to chilling concomitantly with a reduction in oxidative stress, respiration rate and ethylene production, as well as in the expression of ethylene biosynthesis genes, but not of those involved in ethylene perception and sensitivity.

## Introduction

Zucchini (*Cucurbita pepo L*.) can be considered an immature fruit vegetable [1] that is produced and consumed worldwide. The harvest index of immature fruit vegetables, including zucchini, cucumber, eggplant, or green beans, is based principally on size and color, depending upon market needs. For zucchini produced in Spain and consumed in Europe, the fruits have an average length of about 20 cm, just before hardening and darkening of fruit peel, and before undesirable seed development. Immediately after harvesting, the zucchini immature fruit is subject to high rates of water loss and loss of firmness, which contributes to a rapid decrease in postharvest fruit quality and therefore considerable economic loss [1]. Although it is very important to cool the fruit as soon as possible after harvesting, one of the main deterioration symptoms associated with postharvest storage, transportation and marketing is caused by postharvest chilling injury (PCI). Cold storage of zucchini for a minimum of 2–3 days at 4°C induces the appearance of pitting in the fruit surface, and accelerates fruit dehydration and softening [2–6]. The fruit of most current commercial cultivars loses its commercial value when stored at 4°C for less than 7 days [5,6].

To improve the ability of zucchini fruit to withstand cold storage, we are testing different postharvest treatments that may alleviate chilling injury, and identifying different sources of cold tolerance among *Cucurbita pepo* germplasm, which will certainly allow us to develop new PCI tolerant commercial cultivars. The higher cold tolerance of some of the identified cultivars, as well as the positive response of sensitive cultivars to postharvest treatments that alleviate PCI, has been always found to be correlated with a reduction in oxidative stress metabolisms and cold-induced ethylene production [5–7]. Oxidative damage is an early response of cold-sensitive zucchinis to cold exposure [8]. Reactive oxygen species (ROS) triggered by cold storage cause injury to cell membranes by breaking down the double bonds of membrane fatty acids and inducing the accumulation of malonyldialdehyde (MDA), a metabolite that is often used as an indicator of oxidative stress and the degree of structural integrity of the membranes in plants subjected to low temperatures [9,10]. We have reported that zucchini cultivars showing higher tolerance to cold storage suffer fewer PCI symptoms and consequently accumulate lower amounts of reactive oxygen species (ROS) and MDA than those which are more susceptible to cold [5]. Moreover, temperature preconditioning treatment, which induces cold tolerance in fruit, also improves their antioxidant status with lower H_2_O_2_ content and induction of ascorbate peroxidase (APX) and catalase (CAT) activities [7].

Zucchini is a non-climacteric fruit that produces low ethylene at harvest and postharvest. However, postharvest fruit storage at 4°C is able to induce rapidly the production of ethylene upon transfer to 20°C, peaking at 7 days of cold storage [6]. This cold-induced ethylene does not appear to trigger PCI symptoms in zucchini, but has been found to be correlated with chilling sensitivity, less so in the fruit of those cultivars that were more tolerant to PCI, and in response to temperature conditioning treatments that alleviate PCI symptoms [6].

ISW is a passive modified atmosphere packaging (MAP) in which a polymer film with a selective permeability to CO_2_, O_2_, ethylene and water is not applied to shipping containers or retail packages, but rather to individual units of the commodity. When applied to fruit, the selective permeability of the film, and the interplay of the fruit physiology and the physical environment, produces a change in the initial atmospheric conditions to a desirable atmosphere within the package. ISW reduces postharvest losses and extends the shelf life of a number non-climacteric fruits and vegetables such as citrus, pepper and cucumber [11–13], but also some climacteric fruits such as apple and papaya [14,15]. In other climacteric fruits, including tomato and melon, ISW was not fully successful since it enhances undesirable flavor changes or impaired ripening [16,17]. Individual shrink wrapping adds value to fruit and vegetable products, maintaining their freshness for longer because of a reduced moisture loss, chilling injury, firmness and decay, as well as increased protection from mechanical damage during handling and transport. Rao et al. [18] studied the effect of MAP and shrink wrapping on the shelf life of cucumber and reported that shrink wrapping film can extend the shelf life of the immature cucumber fruit for up to 24 days at 10°C. However, the treatment has not yet been tested for the immature fruit of zucchini. In the present paper we have study the effects ISW on different fruit quality parameters in two zucchini cultivars stored for 14 days at 4°C. We found that maintenance of fruit weight and firmness and the reduction of PCI in ISW fruit were accompanied by a reduction in the production of cold-induced ethylene, in the expression of ethylene biosynthesis genes, and in the accumulation of oxidative stress metabolites.

## Material and Methods

### Plant material and experimental design

**Fig 1:**
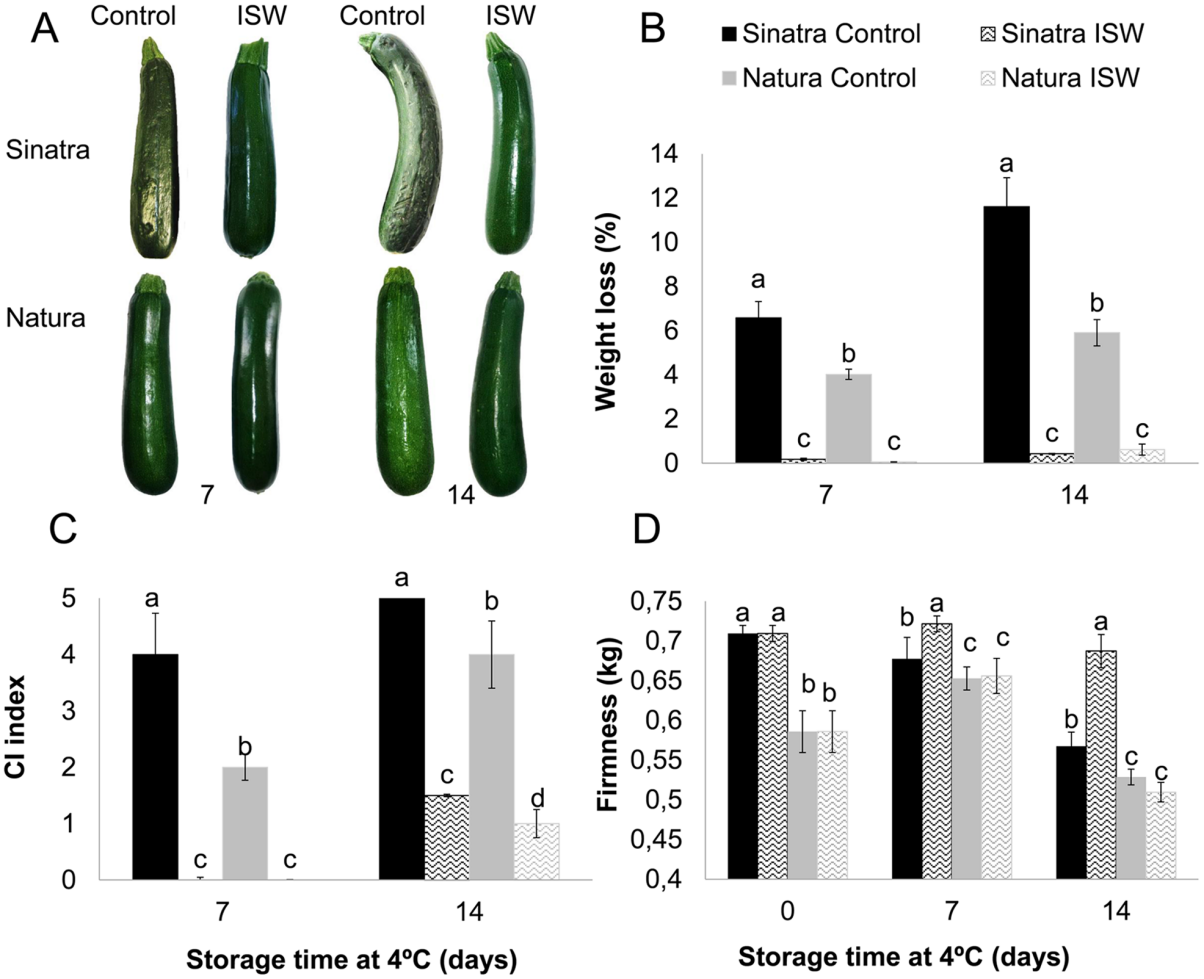
Postharvest quality in control and ISW fruit of Sinatra and Natura zucchini cultivars. (A) fruit pictures (B) weight loss, (C) chilling injury index, (D) firmness. Fruits of the two cultivars were harvested at a similar stage of development, shrink wrapped, stored for 0, 7 and 14 days at 4°C, and then rewarmed at 20°C for 6 hours before unwrapping and taking pictures and performing measurements. Control fruit was left unwrapped for the whole period of time. The results represent the mean and standard deviation of four independent replicates for each sample. Different letters indicate significant differences between samples for each storage time (*p*-value <0.05).

Zucchini fruits (*Cucurbita pepo* L. morphotype Zucchini) were supplied by FRUTAS ESCOBY, S.L. Almería (Spain). The cultivars Natura and Sinatra were used because of their contrasting postharvest behavior during cold storage. The fruit of Natura is more tolerant to chilling injury while the fruit of Sinatra is more sensitive [6] during postharvest shelf life. In order to minimize the effects of pre-harvest management, all fruits were of the same phenological age and were grown in the same greenhouse and harvested simultaneously (Fig 1A). The initial fruit quality and physiological parameters were assessed on a batch of both cultivars. At once the remaining fruits were divided in two groups of 60 fruits per cultivar. The fruits were randomly divided in different groups and then subjected to the postharvest treatments (i) Individual shrink wrapping (ISW) with shrink film microperforated low density polyethylene of 18 μm thickness with the following gas permeability: 7800 ml O_2_ m^-2^d^-1^atm^-1^, 42000 ml CO_2_ m^-2^d^-1^atm^-1^ and 7870 ml C_2_H_4_ m^-2^d^-1^atm^-1^, and (ii) a non-wrapped treatment. Zucchini fruits were individually shrink wrapped by passing through an AWETA sorting and shrink wrapping machine at 280°C for 1,5 s.

Wrapped and unwrapped fruits were stored at 4°C for 14 days. At 7 and 14 days, four replications of 3–4 fruits per cultivar and treatment were transferred to 20°C for 6 hours before sampling and evaluation of quality parameters. Ethylene and CO_2_ production, weight loss, chilling injury index and firmness were measured. Finally, two samples from the homogenated exocarps of each replication were frozen separately in liquid nitrogen and then stored at -80°C for MDA and H_2_O_2_ content and gene expression analyses.

### Ethylene and CO_2_ production

Ethylene and CO_2_ production were determined at 0, 7 and 14 days of storage. Twelve fruits were analyzed for each time, temperature and treatment, i.e. 4 replicates of 3 fruit each. Prior to analysis, the plastic film was removed from ISW fruit, which was then enclosed in sealed 10 litre containers for 6 h at 20°C. After this incubation period, gas samples were taken and ethylene content was determined three times by a gas chromatograph (Varian 3900 GC) fitted with a flame ionization detector (FID). In the same way, CO_2_ was measured three times with Check mate II headspace analyzers (Dansensor). Ethylene production and respiration rates were expressed as nL g^-1^ 6h^-1^ and ml of CO_2_ kg^-1^ 6h^-1^, respectively.

### Evaluation of weight loss, firmness and chilling injury

The percentage of weight loss during storage was assessed by weighing 12 individual fruits at 0, 7 and 14 days after harvesting. The percentage of weight loss of each fruit was calculated according to the following equation:

$$\%\text{ Weight loss }=\;\frac{Wi-Wf}{Wi}x100$$

Where Wi and Wf are initial and final fruit weight respectively.

Fruit firmness was determined by a Stable Micro Systems Texture Analyzer TA.XT-Plus. A 4 mm-diameter probe was used and penetration was conducted at a speed of 1mm·s^-1^ to a depth of 10 mm. Firmness was measured three times in a transversal section in the distal region of the fruit, as this part softens faster during storage.

To assess postharvest chilling injury we measured the surface and severity of PCI symptoms in each fruit after 0, 7 and 14 days of cold storage. The percentage of fruit surface affected by pitting was used to classify each fruit according to the following scale: 0 = no damage, 1≤ 5% damage, 2 = 6–15% damage, 3 = 16–25% damage, 4 = 26–50% damage, and 5 ≥50% damage [6]. To assess the severity of pitting symptoms the scale was 0 = no damage, 1 = very superficial damage, 2 = superficial, 3 = moderate damage, 4 = severe damage, 5 = very severe damage. 12 fruits were analysed for each treatment and storage time. The final PCI index used was the average of both parameters. PCI was assessed at 0, 7 and 14 days of cold storage.

### Determination of malonyldialdehyde (MDA) and hydrogen peroxide contents

MDA content was determined following the procedure described by [5] with some modifications. Exocarp was homogenized in (1:10 w/v) trichloroacetic acid (TCA) and 0.25% (w/v) 2-thiobarbituric acid (TBA) (1:10, w/v), and then heated at 95°C in a water bath for 30 minutes, followed by immediate cooling in ice and centrifugation at 4000 g for 20 minutes at 4°C. The absorbance of the supernatant was measured at 532 and 600 nm. MDA content was expressed as nmol MDA g^-1^ of fresh weight (FW).

The hydrogen peroxide content was determined according to the procedure described by [19]. Exocarp was homogenized with 1% (w/v) TCA (1:10, w/v), and then centrifuged at 12000 g at 4°C for 15 min. Subsequently, 0.5 mL surpernatant was mixed with 0.5 ml 100 mmol potassium phosphate buffer (pH 7) and 2 mL 1 M potassium iodide. After 1 h in the dark the absorbance was measured at 390 nm. The hydrogen peroxide content was expressed as μmol H_2_O_2_ g^-1^ of FW.

### Gene expression analysis by quantitative RT-PCR

Gene expression analysis was performed three times with three replications per time and temperature of storage, and each replication was the result of an independent extraction of total RNA from 3 different fruits. Samples were obtained from fruits stored at different storage temperatures and then rewarmed for 6 hours at 20°C. For each sample, portions of exocarp of 3–4 fruits were homogenized, frozen in liquid nitrogen, and stored at -80°C. Total RNA was extracted from each sample using a Total RNA Mini kit (Bio-Rad). The remains of DNA in RNA samples were eliminated by digestion with RQ1 RNase free DNAse (Promega). Before cDNA synthesis, we verified the absence of DNA in RNA and cDNA samples by PCR amplification with primers for *CpACS4*. cDNA was synthesized from 1 μg of total RNA using iScript reverse Transcription Supermix for RT-qPCR (Bio-Rad). The expression of genes was evaluated through quantitative RT-PCR by using the Rotorgene thermocycler (Qiagen) and iTaq Universal SyBR Green Supermix (Bio-Rad). S1 Table shows the different primers used for q-PCR, which were designed from the 3′ non-coding regions of each gene by using the *Primer Express v 2*.*0* (Applied Biosystem) software. To avoid possible cross-amplification, and before the q-PCR experiments, the size of the PCR products for each pair of primers was tested in agarose gels. Relative expression of each gene was determined by the comparative Ct (*Cycle Threshold*) method using *C*. *pepo Elongation Factor 1-α* (EF-1A) and *Actin* (*ACT*) genes as internal standards. To use this method, we first demonstrated that the efficiency of amplification for each amplicon was roughly equivalent, regardless of the amount of template cDNA. The absolute value of the slope of ΔCt (Ct of the target gene-Ct of the reference gene) versus serial dilutions of cDNA for a given sample must be less than 0.1. The relative expression of each gene to a calibrator sample was calculated using the formula 2-ΔΔCt, where ΔΔCt is the difference between the ΔCt of each sample and the ΔCt of the calibrator sample.

### Statistical analysis

Data were treated for multiple comparisons by analysis of variance (ANOVA), followed by least significant difference test (LSD) with significance level p≤0.05. Sources of variation were storage time and treatment for each cultivar. ANOVA was performed using the statistical software Statgraphic Centurion XVI (STATGRAPHICS. Statpoint Technologies, Inc.,Warrenton, VA). Normality of distribution was verified using Kolmogorov–Smirnov test, when the assumption of normality failed, the variables were transformed. Weather transformation was not possible non-parametric Kruskal–Wallis test was used to compare differences between groups, with significance level at <0.05.

## Results

### Postharvest fruit quality parameters

The ISW packaging treatment greatly improved the postharvest quality and shelf life of the zucchini fruits from two different cultivars, which were subjected to cold storage for 14 consecutive days at 4°C (Fig 1A). One of the most prominent effects was on fruit weight loss. This postharvest parameter increased in the fruits of both cultivars over cold storage, although the performance of Natura fruit, with a weight loss of 4% and 6% at 7 and 14 days of storage, respectively, was much better than that of Sinatra, which lost 7% and 13% at the same storage times (Fig 1B). As expected, ISW treatment almost abolished the dehydration of zucchini fruit and therefore the percentage of weight loss was reduced to almost 0% in both varieties (Fig 1B).

More interestingly, ISW reduced the occurrence of surface pitting of both cultivars (Fig 1C). Sinatra fruit proved more sensitive to PCI than Natura. At 7 days of storage, the Natura fruit showed an average PCI index of 2, while that of Sinatra reached a value of nearly 4 (Fig 1C). At 14 days of storage the average PCI index of Sinatra fruit was still higher than that of Natura, but the fruit of both cultivars was so affected that it had already lost its commercial value (Fig 1C). In ISW fruit of the two cultivars PCI symptoms were not detected at 7 days of storage, and were considerably reduced in comparison to control fruit after 14 days of storage (Fig 1C). In fact, at 14 days ISW fruit had lower PCI symptoms than control fruits at 7 days of storage (Fig 1C). These data demonstrate that the ISW packaging limited the advance of PCI symptoms, extending fruit shelf life for at least 7 days at 4°C.

The response of fruit firmness to ISW treatment depended on the cultivar (Fig 1D). In the more sensitive cultivar, Sinatra, this treatment considerably reduced the percentage of firmness loss at 7 and 14 days of cold storage, even leading to a slight increase in fruit firmness at 7 days. The firmness of Natura fruit was higher than that of Sinatra during the whole storage period, but it did not respond to ISW treatment (Fig 1D).

### Ethylene production and ethylene gene expression

**Fig 2:**
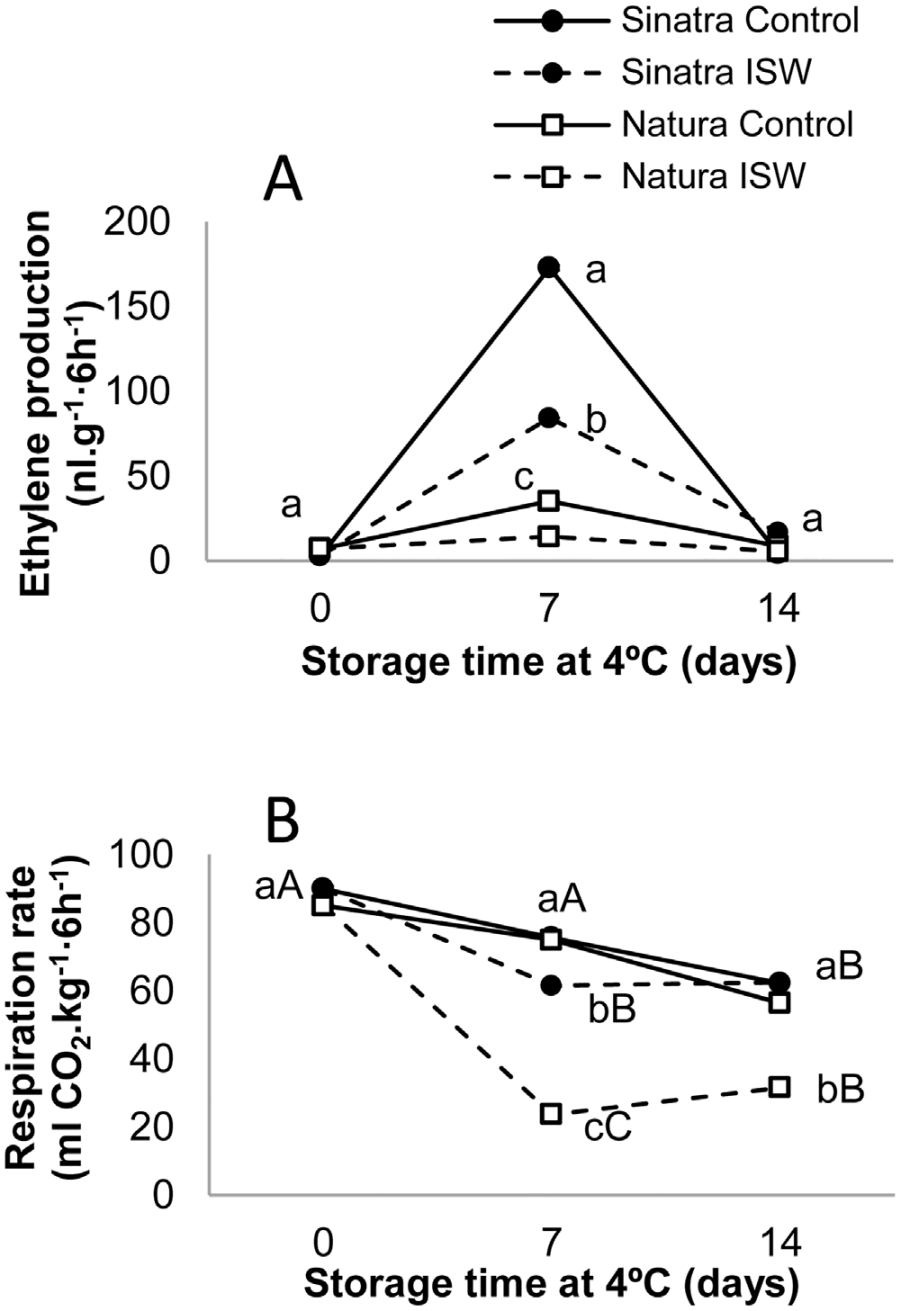
Evolution of ethylene (A) and CO_2_ (B) in control and ISW refrigerated fruit of Sinatra and Natura. Control and ISW fruit was stored for 0, 7 and 14 days at 4°C and then rewarmed at 20°C for 6 hours before measurements. The results represent the mean of four independent replicates for each sample. Capital letters indicate differences between storage time for each cultivar and treatment, while lowercase letters indicate differences between cultivars and treatments at each storage time (*p*-value <0.05).

The longer shelf life and higher quality of ISW fruit was found to be correlated with a reduction in the production of ethylene (Fig 2A). At harvest the zucchini fruit of both cultivars produced no ethylene, which demonstrates the non-climacteric nature of this immature fruit. In accordance with previous results [6], seven days of cold storage induced the production of ethylene in fruit upon rewarming at 20°C for six hours (Fig 2A), and cold induced ethylene is correlated with chilling sensitivity, being much higher in the cold sensitive cultivar Sinatra [6] (Fig 2A). The fruit of the more tolerant cultivar Natura, in fact, hardly induced the production of ethylene after 7 days of cold storage (Fig 2A). After 14 days of cold storage, although the fruit were rewarmed at 20°C, ethylene production returned to its original low value (Fig 2A). In the fruit of Sinatra, ISW significantly reduced the production of ethylene after 7 days of cold storage, and in the fruit of Natura, the production of ethylene remained very low in control and treated fruits for the complete storage period (Fig 2A).

**Fig 3:**
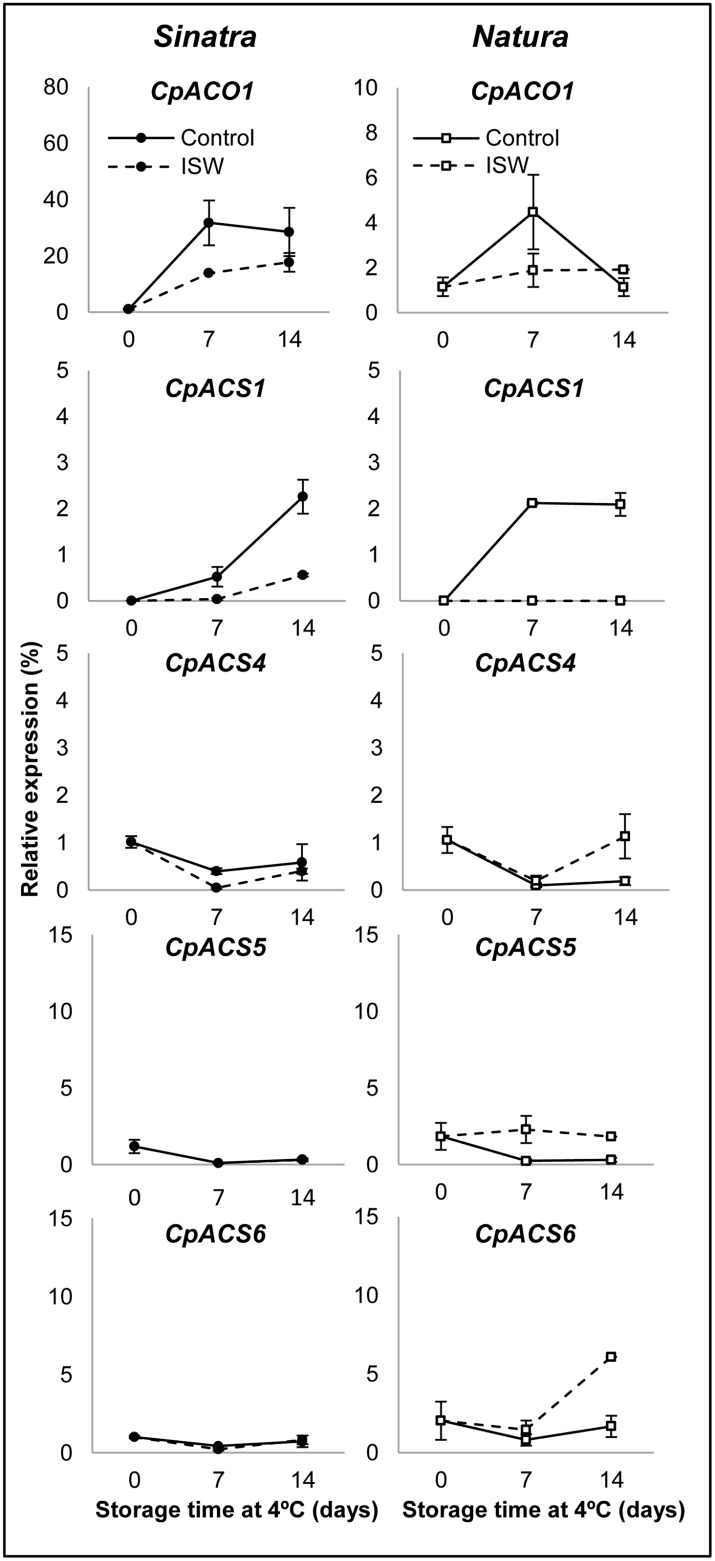
Expression profiles of ethylene biosynthesis genes in control and ISW fruit of Sinatra and Natura. Control and wrapped fruits of the two cultivars were stored for 0, 7 and 14 days at 4°C and then rewarmed at 20°C for 6 hours before collecting the exocarp material that was used for gene expression analyses. Transcript levels for each gene were assessed by quantitative RT-PCR. Data were standardized with the expression at harvest. Results represent the mean and standard deviation of three independent replicate samples for each cultivar, treatment and storage time.

**Fig 4:**
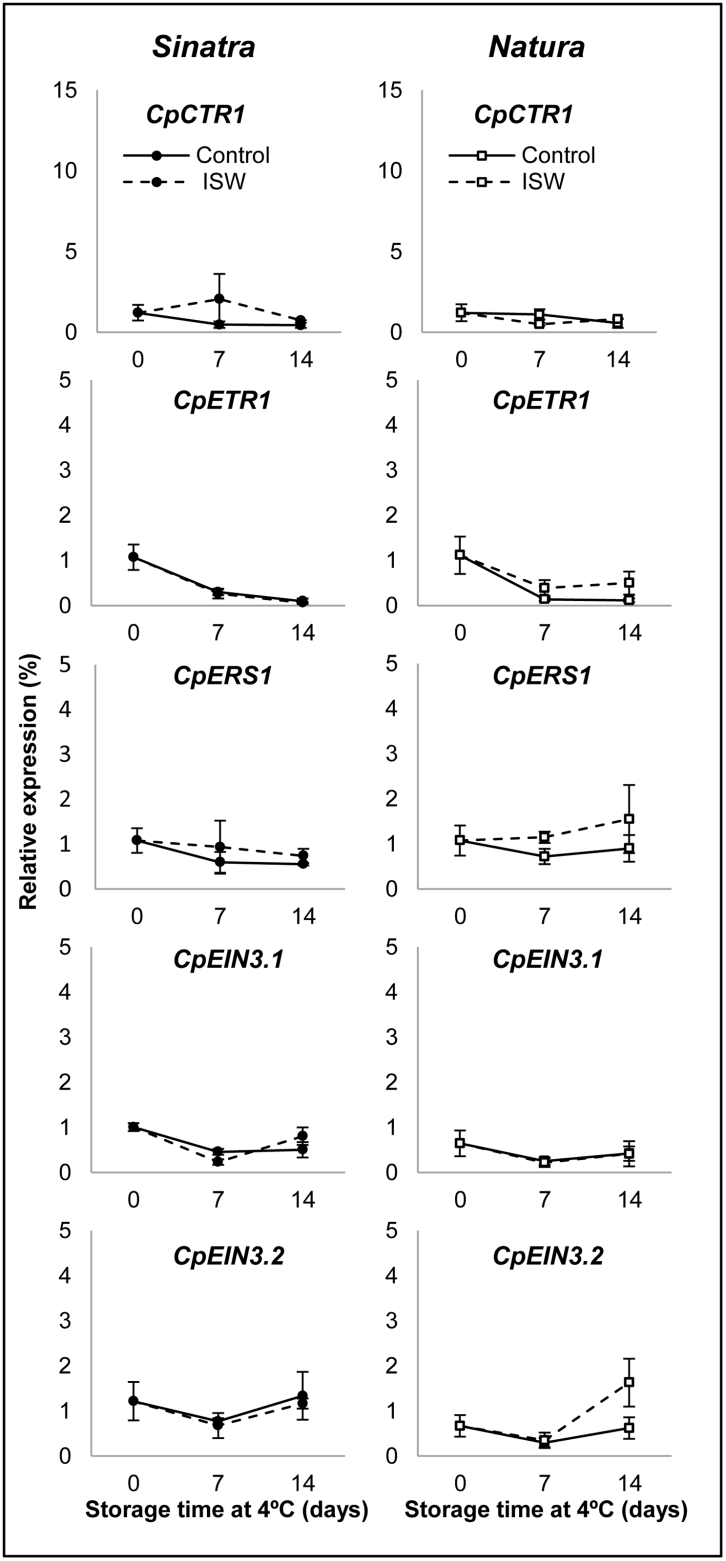
Expression profiles of ethylene perception and signaling genes in control and ISW fruit of Sinatra and Natura. Control and wrapped fruits of the two cultivars were stored for 0, 7 and 14 days at different temperatures and then rewarmed at 20°C for 6 hours before colleting the exocarp material used for gene expression analyses. Transcript levels for each gene were assessed by quantitative RT-PCR. Data were standardized with the expression at harvest. Results represent the mean and standard deviation of three independent replicate samples for each cultivar, treatment and storage time.

To analyze the molecular mechanisms underlying ethylene production and response during zucchini cold storage we have studied the expression of 13 genes involved in the biosynthesis, perception and response to ethylene in control and ISW fruit of Natura and Sinatra. Genes were selected based on *C*. *pepo* transcriptome [20] and previous gene expression data in zucchini [21,22] and Figs 3 and 4 show the expression profiles of genes involved in the biosynthesis or the response to ethylene, respectively. The transcripts of three of a total of eight ethylene biosynthesis genes studied in this paper *(CpACS2*, *CpACS3* and *CpACS7*) were not detected in the fruit under our postharvest conditions (data not shown). However, two of the biosynthesis genes (*CpACO1* and *CpACS1*) were highly upregulated in the fruit of both cultivars in response to cold storage (Fig 3), which suggests that these two genes are involved in cold-induced ethylene production. In ISW fruit, the induction of *CpACO1* and *CpACS1* was reduced or completely suppressed in comparison with control fruits. The expression level of the other three biosynthesis genes (*CpACS4*, *CpACS5* and *CpACS6)* was very low and varied slightly in response to cold and ISW treatments (Fig 3). Only in ISW fruit of Natura was there a slight increase in the accumulation of *CpACS4*, *CpACS5* and *CpACS6* at 14 days of cold storage in comparison with control fruit (Fig 3). Since at 14 days of storage the observed ethylene production was very low (Fig 2A), it is likely that this induction of *ACS* in ISW fruit was not accompanied by an induction of *ACO* genes.

The ethylene perception and signaling genes *CpERS1*, *CpETR1*, *CpCTR1*, *CpEIN3*.*1* and *CpEIN3*.*2* barely changed in response to cold storage or ISW (Fig 4), indicating that ethylene perception and signaling does not appear to play an important role in the response of zucchini fruit to ISW packaging or cold storage in either Sinatra or Natura (Fig 4). Only the gene *CpACS3*.*2* was slightly induced in the ISW fruit of Natura at 14 days of cold storage, but not in that of Sinatra (Fig 4).

### Respiration rate and oxidative stress

Respiration rate was measured at harvest and after cold storage. In the latter case, prior to measurement the plastic film was removed from the wrapped fruits and all fruits were rewarmed at 20°C for 6 hours. At 7 days of cold storage, control fruit maintained their respiration rates during postharvest storage at 4°C in both Sinatra and Natura, but these rates fell after 14 days of cold storage (Fig 2B). In the ISW fruit of both cultivars, but especially in that of Natura, the production of CO_2_ fell significantly, with a fourfold reduction in Natura fruits in comparison with control fruits (Fig 2B).

**Fig 5:**
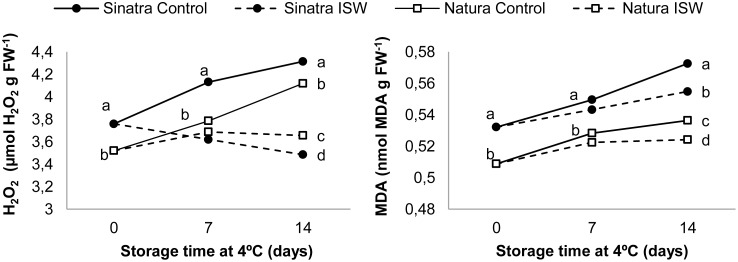
Contents of H_2_O_2_ (A) and MDA (B) in control and ISW fruits of Sinatra and Natura cultivars, stored at 4°C. Samples were collected after rewarming fruit at 20°C for 6 h. The results represent the mean of four independent replicate samples for each cultivar, storage time and treatment. Different letters indicate significant differences between sample means at each specific storage time (*p*-value <0.05).

To determine ROS accumulation and membrane damage in control and ISW fruits, we measured the contents of hydrogen peroxide and MDA, the latter being a product of lipid peroxidation and membrane integrity. The results of control and ISW fruits from Sinatra and Natura stored at 4°C at 0, 7 and 14 days are shown in Fig 5. In control fruit of both cultivars, hydrogen peroxide was accumulated upon cold storage, although its content was always significantly higher in the fruit of the more sensitive cultivar Sinatra (Fig 5A). On the other hand, the hydrogen peroxide content remained stable or even fell in ISW fruit of Natura and Sinatra, respectively, with significant differences between control and ISW fruit at 7 and 14 days of cold storage in both cultivars (Fig 5A).

MDA content increased throughout postharvest zucchini fruit storage at 4°C in both control and ISW fruit of the two cultivars, but with significantly higher content in the more sensitive cultivar Sinatra at all three storage times (Fig 5B). ISW fruit reduced the accumulation of MDA with respect to control fruit in both cultivars, although the MDA content in ISW fruit of Sinatra was always higher than in those of Natura at the storage times analyzed (Fig 5B).

## Discussion

### ISW enhances the quality of zucchini fruit subjected to cold storage

Cold storage is a postharvest technology that preserves the quality and therefore increases the shelf life of many fruits and vegetables. There are, however, a number of tropical and subtropical fruits that are very susceptible to cold during postharvest, developing PCI and accelerating loss of weight and firmness, and decay when stored at below optimum temperatures. The immature fruit of zucchini is extremely susceptible to cold storage and PCI [3,4,23], although variations for this trait have been detected among commercial and traditional cultivars of this crop [5,6]. The fruit of the two commercial hybrids analyzed in this paper, Natura and Sinatra, showed differences in their sensitivity to PCI, the former proving a more cold-tolerant genotype than the latter [6]. In correlation with PCI index, based on the percentage and severity of pitting symptoms on the fruit surface, Natura control fruit also showed a lower loss of weight and firmness (Fig 1), confirming its greater tolerance to cold storage.

Our data demonstrate that ISW packaging technology applied to zucchini was not only able to reduce weight loss resulting from the dehydration of immature fruit, but also to reduce significantly firmness loss and the percentage and severity of pitting symptoms on the fruit surface of two zucchini cultivars. The treatment also delayed the onset of PCI in the fruit of the two cultivars, retaining their harvest fresh surface appearance even after 14 days of cold storage. Under the same storage conditions, however, the control fruit of Sinatra and Natura lost their commercial value at 7 and 14 days of cold storage, respectively, essentially because of the percentage of fruit surface covered by PCI. These results indicate that ISW is able to enhance cold-tolerance in zucchini, extending the shelf life of cold stored fruit for more than 7 days. This was especially noteworthy in the more sensitive cultivar Sinatra, in which ISW fruit suffered no symptoms of PCI nor loss of weight and firmness at 7 days of cold storage, while at 14 days their loss of quality parameters was less than 10% that of control fruit (Fig 1). We have found a slight increase in firmness of Natura fruit during cold storage. Whether this slight increase is a result of lignification, as has been observed in the fruit of different loquat cultivars [24,25], needs to be further evaluated. In Natura, ISW treatment also reduced weight loss and PCI, although no significant differences were detected for firmness between wrapped and unwrapped fruit throughout the storage time (Fig 1). This is the first report indicating the benefits of ISW packaging in zucchini, but similar trends in weight loss, firmness and PCI have been reported for ISW cucumbers [13,26], also an immature fruit of the *Cucurbitaceae* family, as well as in other fruits and vegetables [11,15, 27–29].

The minimum loss of freshness and firmness and the reduced PCI of shrink wrapped zucchinis may be a result of maintaining high relative humidity, produced by the limitation of water vapour diffusion and higher vapour pressure inside the package, and by the modified atmosphere (MA) around each piece of fruit. An increase in water vapor pressure around the fruit concomitant with a decrease in the transpiration rate has also been observed in other fruit and vegetable under plastic film wrapping, including broccoli [28], apricot [30], sweet cherry [31] and loquat [32].

In zucchini, treatments involving reduced concentrations of O_2_ or high concentrations of CO_2_ before cold storage at 2.5°C have been reported as effective in reducing PCI and in increasing polyamine levels [3], and chilling tolerance has always been found to be associated with a reduced respiration rate in refrigerated fruit [23]. Atmospheres with low O_2_ (1–5%) and high CO_2_ (5–10%) concentrations are able to extend the shelf life of fresh fruits and vegetables because of a reduction of the respiration rate due to substrate depletion, and a restriction of ethylene biosynthesis [33]. Temperature is also known to be one of the main external factors regulating the respiration rate of fruit [34]. Although we could not measure the evolution of O_2_ and CO_2_ inside individual packages, the gas selectivity and permeability of the film used (see Material and Methods) ought to be effective to reduce O_2_ and to increase CO_2_ within an acceptable range for zucchini conservation. In fact, in ISW fruit not only were fruit quality parameters retained, on opening the wrapping no symptoms of bad aroma or off-favours were detected, which suggests no anaerobic metabolism or accumulation of either ethanol or acetaldehyde [35]. The observed reduction in the respiration rate of ISW fruit (Fig 2) might therefore be due to a combination of low temperature of storage and modified atmosphere in the individual bags. Previous research works have also detected a reduced respiration rate in fruit and vegetables such as broccoli [28], or tomato [36] subjected to ISW, in plum, and cucumber fruits exposed to MAP [37,38], as well as in plum, peach and pomegranate protected by edible coatings [39,40]. This reduction in the respiration rates of fruits could be due to exposure to high concentrations of CO_2_ and low concentrations of O_2_ in the individual bags [41,42]. In the present work, Natura fruits showed a higher reduction in respiration rate than Sinatra ones, also indicating that in zucchini this regulation is genotype dependent and has a far greater effect on ISW fruit of the more cold tolerant cultivar. These differential responses may be linked to differences in low temperature response between the two cultivars.

### ISW inhibits the production of ethylene and the expression of ethylene genes

In a previous paper we reported that the storage of zucchini fruit at low temperatures induces the production of ethylene once the fruit is rewarmed for a few hours at 20°C. As shown in Fig 2, this chilling-induced ethylene peaks at 7 days of cold storage plus 6 h of rewarming, and is higher in Sinatra, the cultivar showing higher sensitivity to PCI. Our results agree with previous data showing that chilling-induced ethylene is correlated with chilling injury and chilling sensitivity in zucchini [6]. In accordance with temperature preconditioning treatments, which also alleviate PCI in zucchini, the ISW treatment significantly inhibited chilling-induced ethylene in the more sensitive cultivar. In the more tolerant genotype Natura, this reduction was not obvious due to the very low level of ethylene production in the refrigerated fruit of this cultivar (Fig 2). This inhibition of ethylene production promoted by ISW was previously reported in the climacteric papaya [15], but also in the non-climacteric fruit of pepper [29], and the immature fruit of cucumber [13]. MAP has been also found to reduce the production of ethylene in diverse fruit such as plum and apple [37,43].

The role of ethylene in the onset of PCI is unclear. Since many cold sensitive fruits produce ethylene concomitantly with the development of PCI [44], it has been speculated that this ethylene could be involved in the development of PCI [45]. Nevertheless this ethylene could also be the consequence of cold stress and result from PCI in cold sensitive fruits. Indeed, in zucchini we have recently reported that this chilling-induced ethylene was not necessary for the onset of PCI, but rather a cold induced response [6]. Despite this, the inhibition of ethylene production observed in ISW fruit again demonstrates that there is a correlation between chilling-induced ethylene and chilling sensitivity in zucchini, and that the reduction of ethylene in cold stored fruit was always associated with a reduction in PCI and therefore with a higher tolerance to cold storage. As proposed previously, this chilling induced ethylene may therefore be used as an earlier marker of the tissue that may show the secondary, downstream visual symptoms of PCI [6]. The gene expression data also support this conclusion, as many of the upstream regulatory genes in the ethylene signal transduction pathway did not change under cold, but those catalyzing steps in ethylene production did.

Even less is known about the molecular mechanisms associated with ethylene production and response as a result of the postharvest storage of zucchini fruit at low temperatures. The biosynthesis of ethylene comprises two steps: S-adenosymethionine is converted to 1-aminocyclopropane-1.carboxylate (ACC) by the limiting enzyme ACC synthase (ACS), and ACC is then converted to ethylene by ACC oxidase (ACO), and the genes coding for these two enzymes belong to multigenic families in plant genomes [33]. In this paper we have studied the expression profiles of one *ACO* and seven *ACS* genes during the storage of zucchini fruit at 4°C for 14 days and in response to ISW packaging. The results indicate that the promotion of ethylene biosynthesis in chilled fruit depends mainly on two of the analyzed genes, *CpACS1* and *CpACO1*, whose expressions were highly induced in control fruit under low temperature storage, and were downregulated in response to ISW. The transcripts of other *ACS* genes such as *CpACS2*, *CpACS3* and *CpACS7* were not detected in the fruit during postharvest, and those of the genes *CpACS4*, *CpACS5* and *CpACS6* were not regulated by either cold or ISW packaging in the cold sensitive cultivar Sinatra, although they were slightly upregulated in the ISW fruit of the more tolerant cultivar Natura (Fig 3). The regulation of *ACS* and *ACO* genes in response to chilling is not constant among different fruits. Thus *ACS* and *ACO* genes have been found to be either upregulated or downregulated in response to cold storage in papaya [46], citrus [47], banana [48] or peach [49]. We have found that *CpACS1* and *CpACO1* are more sensitive to low temperature, and that the reduction of ethylene production promoted by ISW treatment was correlated with a downregulation of these two genes. The genes *CpACO1* and *CpACS1* would therefore be the perfect biotechnological targets to improve cold tolerance. In fact, we have already started the identification of loss-of-function mutants for these two genes in an EMS mutant collection of zucchini.

None of the five ethylene perception and response genes analyzed in this paper was found to be significantly modulated by either cold storage or ISW packaging during the postharvest storage of zucchini fruit. The perception genes *CpETR1* and *CpER1*, and the signal transduction genes *CpCTR1* and *CpEIN3*.*1*, did not change their expression in response to the treatment. Only the gene *CpEIN3*.*2* was slightly upregulated in the ISW fruit of the cultivar Natura (but not in those of Sinatra) after 14 days of cold storage (Fig 4). In other systems, including both climacteric and non-climacteric fruits, the expression of ethylene receptors and ethylene signal transduction genes in response to cold storage varied greatly and depended on the isoform analyzed. In the climacteric papaya, the expression of *ETR1*, *ERS1* and *CTR1* was not induced by cold storage, but the expression of *CTR2*, *EIN3*.*1a* and *EIN3*.*1b* was induced after 20 days of cold storage [46]. Ethylene perception and signaling genes were also differentially regulated in the climacteric fruits tomato, kiwi and pear [50–55]. In non-climacteric fruit, such as that of grapefruit and loquat, ethylene receptors and response genes were also differentially upregulated by cold storage [56,57], although in these cases ethylene is not required for fruit ripening. Taken together these results indicate that chilling is able to induce some isoforms of ethylene biosynthesis, perception and response genes in both climacteric and non-climacteric fruit. In non-climacteric fruit the induction of these genes is not associated with fruit ripening but should rather be associated with fruit response to cold stress. The regulation of specific ethylene perception and response genes such as *EjETR1* and *EjEIL1* in loquat, has suggested that they may be involved in PCI development [57]. Our results in zucchini suggest that certain ethylene biosynthesis genes, but not all of them, participated in the fruit response to chilling temperature and ISW treatment, but that ethylene perception and signal transduction pathway genes do not appear to be involved in PCI development of control fruit, nor in the reduction of PCI symptoms as a result of ISW packaging.

### ISW inhibits oxidative stress and oxidative damage

It has been widely reported that the storage of fruits at low temperatures is able to induce oxidative stress, leading to an increase in the production of reactive oxygen species (ROS) causing progressive oxidative damage, such as lipid peroxidation of both cellular and organelle membrane that aggravates oxidative stress through the production of lipid-derived radicals [8–10,58]. The accumulation of MDA, one of the final products of the peroxidation of unsaturated fatty acids in phospholipids, is frequently used as a marker of membrane oxidative damage. To combat this oxidative stress, plants trigger various enzymatic and non-enzymatic antioxidant defense mechanisms. Oxidative damage to the tissues will therefore depend on a delicate equilibrium between ROS production and the antioxidant defense mechanisms that operate in the cells scavenging for ROS overproduction [58].

In zucchini, it has been reported that the storage of fruit at low temperatures induces changes in both the accumulation of ROS and in the activity of antioxidant enzymes such as catalases, ascorbate peroxidases and superoxide dismutases [5,7,8,59]. Postharvest treatments that have been shown to induce chilling tolerance in zucchini, including temperature preconditioning treatments [7,8] and external applications of polyamines [59], were able to induce the enzymatic oxidative defense mechanisms while reducing the content of H_2_O_2_ and MDA. Changes in carbohydrate content in zucchini fruit under low temperature have been related not only with the importance of soluble carbohydrate as osmoprotectants and stabilizers of cell membranes but also with their role as ROS scavengers [60]. Our results in this paper also indicate that the success of ISW, the most effective current treatment to induce cold tolerance in zucchini, is also concomitant with a reduction in oxidative stress induced by chilling. The treatment not only reduced the production of H_2_O_2_ throughout the storage period in both Natura and Sinatra, but also diminished the accumulation of MDA, which manifested itself in a reduction of cell damage. The manipulation of this oxidative stress and oxidative damage is therefore essential to reduce PCI symptoms and to increase the postharvest quality of zucchini fruit under cold storage.

In conclusion, individual shrink wrapping of zucchini fruit with a selective film during postharvest was able to induce cold tolerance in two cultivars showing differences in chilling sensitivity, by considerably reducing chilling injury and the loss of weight and firmness during the storage period at 4°C. This improvement in fruit quality parameters was associated with a reduction in the production of ethylene and a downregulation of ethylene biosynthesis genes *CpACS1* and *CpACO1*, together with a reduction in the respiration rate of fruit and the inhibition of oxidative stress and oxidative damage processes.

## Supporting Information

S1 Table Primers used in quantitative real time RT-PCR reactions. ^1^ *Cucurbita pepo* unigenes available at http://www.cucurbigene.net.
(DOCX)
