# Supplementary material for: Individual Shrink Wrapping of Zucchini Fruit Improves Postharvest Chilling Tolerance Associated with a Reduction in Ethylene Production and Oxidative Stress Metabolites
Source: PLoS One. 2015 Jul 15;10(7):e0133058. doi: 10.1371/journal.pone.0133058 (PMC4503597; doi:10.1371/journal.pone.0133058)
Supplement: S1 Table — 1 Cucurbita pepo unigenes available at http://www.cucurbigene.net. (DOCX) [file pone.0133058.s001.docx]

| Gene ^1^ | Reference  accession number | Q-PCR primer sequences |
| --- | --- | --- |
| *CpEF*  *CpACTIN*  *CpACO1*  *CpACS1*  *CpACS2*  *CpACS4*  *CpACS5*  *CpACS6*  *CpACS7*  *CpCTR1*  *CpETR1*  *CpERS1*  *CpEIN3.1.1*  *CpEIN3.1.2* | Obrero et al.,2011  HO702383  Olimpieri et al.,2007  HM594170  Unpublished  HQ847860  Sato et al., 1991  AAA331133  Blanca et al., 2011  CUTC0483191  Blanca et al., 2011  CUTC036131  Blanca et al., 2011  CUTC0383771  Blanca et al., 2011  CUTC0406831  Martínez et al., 2013  KC662095  Manzano et al, 2010b  FJ917748  Manzano et al., 2013  JX014243  Manzano et al., 2013  JX014245  Martínez et al., 2013  KC620381  Martínez et al., 2013  KC620381 | GCTTGGGTGCTCGACAAACT  TCCACAGAGCAATGTCAATGG  CCTCTCAATCCCAAAGCTAACAG  CGGCCTGGATAGCAACATACA  CATTTGGGACAAAGGTGAGTAACTAC  GCTCGGAGACCCTTGATCAA  TGTCACTGAGCCAGGTTGGTT  GCAACGTCAACGGTGTTGTC  ACAAGGAGGTAGCCAAGGTTAAG  CCCACGACTCAGTTAAACTTTTG  CAGCAGCTTCTCCCATAATTCTA  CGTTAATGAATACCTGGAGACGA  TGTGCATCTGAAAGCATAGAAGA  TTAGAAGATAGCGACCGAGACAG  GAAACCAGCTACCAAGAGAAACA  TTTTCGTTCGAAGAGTAGATTG  GGTTTAGAGTCGGAGCGATTTAT  CTCTCACCAGCATTTTCTGTCTT  ACTAATGATTAGTTGTGGCTGGATACA  AACAGTAATGACATTTCCTAGCAAACA  AAAGGAGAGCTGCCTGAGAGTC  CACGACGCTCTATAAGTTCCGA  GAGCGTCGGGTTCTATTCGA  AACCTGGGATATGCCTTGTATGTTAC  GCCATCAAATGTTCACAAGACCG  AATGAAAAGAGTGCAAGCAGTCTTAC  TAGCAGCCAATTCAACCAGTTTAAGCC  CGGTAAAGCATCGAATTGAGATCAGG |
